# Data on the influence of TiN on wear and corrosion behavior of Ti–6Al–4V alloy fabricated through spark plasma sintering

**DOI:** 10.1016/j.dib.2018.06.049

**Published:** 2018-06-22

**Authors:** F.M. Kgoete, A.P.I. Popoola, O.S.I. Fayomi

**Affiliations:** a Department of Chemical, Metallurgical & Materials Engineering, Tshwane University of Technology, P.M.B X680, Pretoria 0001, South Africa; b Department of Mechanical Engineering, Covenant University, P.M.B X1034, Ota, Nigeria

## Abstract

Data about bulk properties of Ti–6Al–4V based composites specimen achieved by powder metallurgy route using spark plasma sintering (SPS) technique is presented, with focus on the effect of TiN particles on wear and corrosion behavior of the resultant composites. Two microsized kind of powders are combined; Ti–6Al–4V and TiN. The powder mixing and SPS processing has been enhanced and consolidated.

**Specifications Table**

**Table:** 

Subject area	*physics*
More specific subject area	*Powder Metallurgy*
Type of data	*Table, images, graph, figure*
How data was acquired	*SPS (FCT Systeme GmbH Rauenstein), hardness (Emco Test Dura scan Microhardness tester), SEM (JEOL-JSM-7600F Field Emission Scanning Electron Microscope), Corrosion (Autolab PGSTAT 101 Metrohmpotentiostat), PerkinElmer Thermal Gravimetric Analyser (TGA 4000), wear (Anton Paar Wear Tester).*
Data format	*Examined data*
Experimental factors	*Data was attained from spark plasma sintered composites. The powders were tubular mixed for 4 h subsequent to spark plasma sintering process.*
Experimental features	*Following to polishing, compacts were experimented through SEM-EDS,Anton paar wear tester, XRD, hardness and corrosion tests were done to determine the mechanical, corrosion and wear properties of the spark plasma sintered composites.*
Data source location	*Tshwane University of Technology Laboratory, Pretoria,South Africa*
Data accessibility	*All the data are in this data article.*

**Value of the data**

• This data could be used to further improve wear and corrosion properties of Ti–6Al–4V alloy for various applications including aerospace.
• The data could be used to determine the optimal TiN addition necessary to achieve enhanced properties of titanium made components.
• The data could be used to develop stable spark plasma sintered Ti–6Al–4V based composites which can be employed in corrosion related industries.
• Results can be stretched to other varying ceramic particulates not discussed in this paper.

## 1 Data

The data article provides the effect of varying titanium nitride (TiN) additions on microstructure, corrosion and wear properties of Ti–6Al–4V alloy fabricated through powder metallurgy route; by spark plasma sintering technique [1].

## 2 Experimental design, materials, and methods

### 2.1 Data collection

Microsized Ti–6Al–4V–*x*TiN powders have been blended via spark plasma sintering method [1], [4]. Density measurements, hardness, corrosion, SEM-EDS, and XRD data of the samples are presented. The wear properties of the fabricated specimen are presented.

### 2.2 Data analysis and presentation

Microsized titanium powder (Ti–6Al–4V) of (45–90 µm particle spherical, from TLS Technik GmbH) and titanium nitride powder (TiN) of (<3 µm particle size from sigma Aldrich) were provided and mixed according to the chemistry proportions, as recorded in Table 1, and the powders were considered in different quantities as presented in Table 2.

**Table 1:** Starting materials.

**Powder**	**Particle size (µm)**	**Density (g/m^3^)**	**Purity**
Ti–6Al–4V alloy	>45	4.43	>99
Titanium Nitride	<3	5.40	>99

**Table 2:** Properties of sintered Ti–6Al–4V and Ti–6Al–4V–TiN composites at 1000 °C.

**Sample**	**Measured density (cm^3^)**	**Theoretical density (g/m^3^)**	**Relative density (%)**	**Porosity (%)**	**Sintering temperature (**°**C)**
Ti–6Al–4V Alloy	4.369587	4.43	98.6	1.4	1000
Ti–6Al–4V–5TiN	4.393734	4.47	98.3	1.7	1000
Ti–6Al–4V–10TiN	4.380074	4.511	97.1	2.9	1000
Ti–6Al–4V–15TiN	4.379732	4.553	96.2	3.8	1000

Three samples with varying titanium nitride amounts from 5–15 wt% were set and mixed in a tubular mixer preceding to further process. Spark plasma sintering method using SPS FCT Systeme GmbH Rauenstein model was employed [1], [2], [4]. Ideal operational parameters were used. Sintering temperature was 1000 °C, pressure 50 MPa and the holding time 6 min under argon atmosphere [3].

**Fig. 1:**
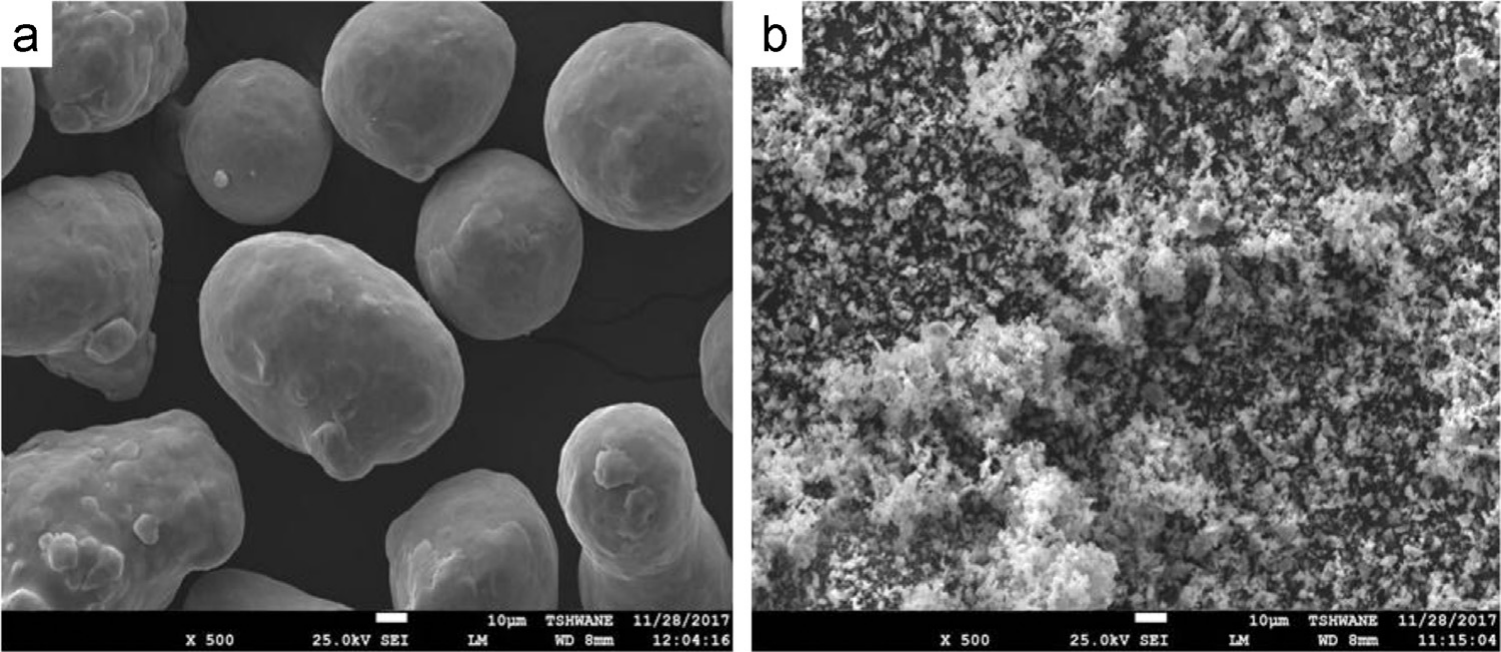
SEM photographs of starting materials. (a) Ti–6Al–4V and (b) TiN.

**Fig. 2:**
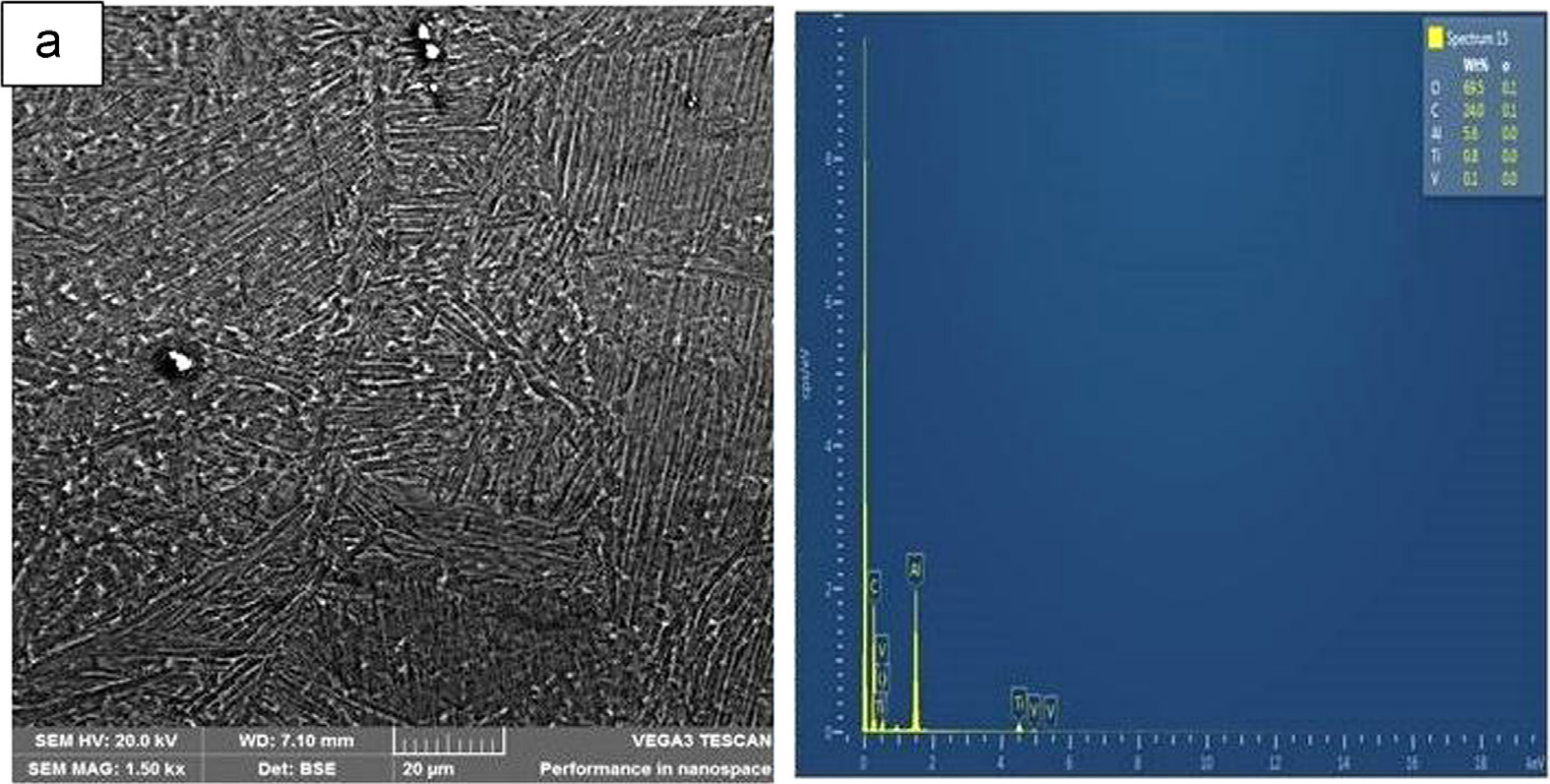
SEM-EDS analysis of Spark plasma sintered Ti–6Al–4V Alloy.

Fig. 1(a) and (b) displays the SEM morphology of Ti-6Al-4V and titanium nitride powders and the microstructural observation are illustrated in Fig. 2(a)–(d).

**Fig. 3:**
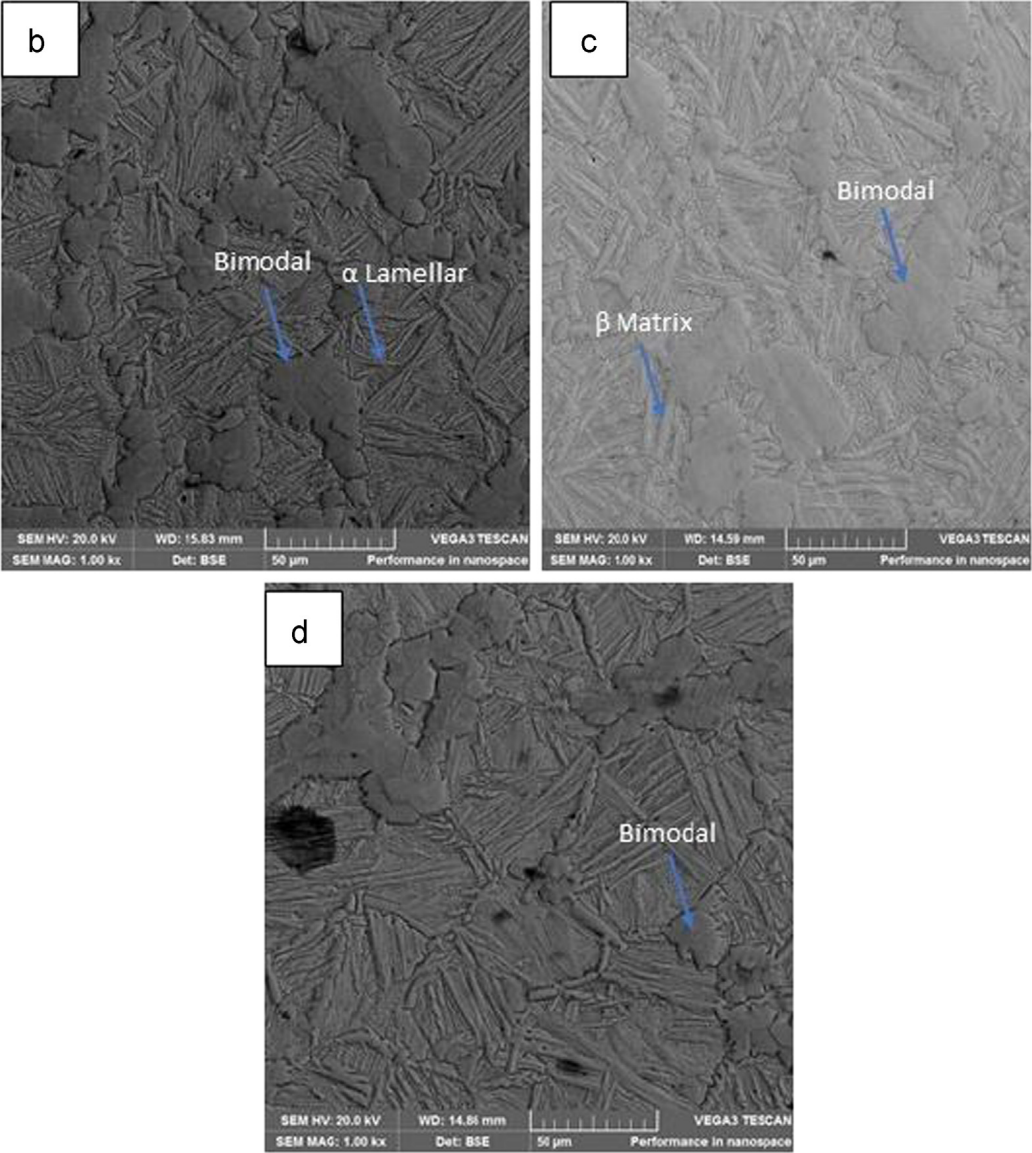
Backscatter SEM photographs of the fabricated. (b) 95Ti–6Al–4V–5TiN, (c) 90Ti–6Al–4V–10TiN, and (d) 85Ti–6Al–4V–TiN.

Fig. 2(a) displays the SEM-EDS of the spark plasma sintered Ti-6Al-4V alloy. The morphology of the reinforced Ti–6Al–4V alloy composites are revealed in Fig. 3(b-d) [6].

**Fig. 4:**
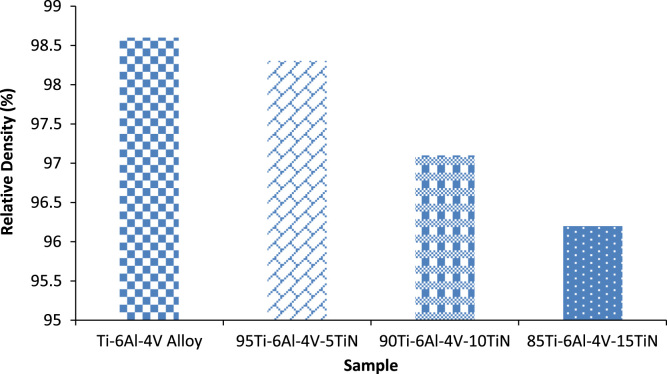
Relative densities of the sintered compacts of Ti–6Al–4V and developed Ti–6Al–4V–*x*TiN.

Fig. 4 illustrates relative densities of the sintered compacts [6].

**Fig. 5:**
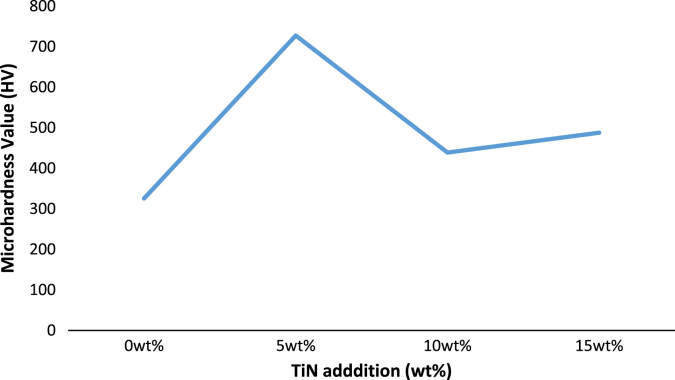
Hardness trend with and without TiN content for Ti–6Al–4V based composites.

Microhardness trend of the spark plasma sintered compacts can be observed in Fig. 5.

**Fig. 6:**
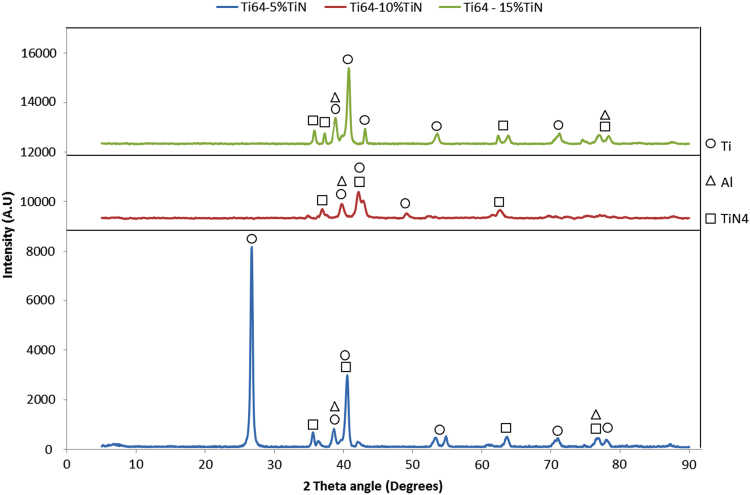
XRD diffractogram of Ti–6Al–4V–*x*TiN.

Fig. 6 shows the XRD patterns of Ti–6Al–4V alloy obtained from spark plasma sintering of with and without TiN at the sintering temperature of 1000 °C and holding time of 6 min.

**Fig. 7:**
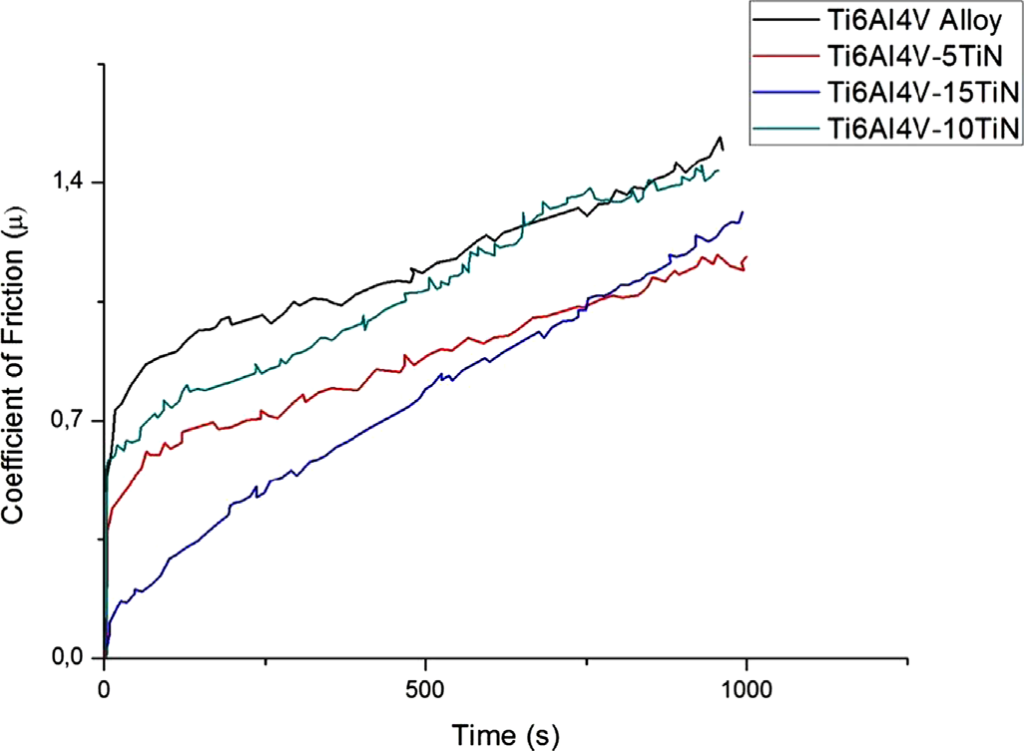
Variations of the coefficient of friction with time of Ti–6Al–4V–TiN binary spark plasma sintered composites.

Fig. 7 shows coefficient of friction traces for Ti–6Al–4V and Ti–6Al–4V–*x*TiN composites.

**Fig. 8:**
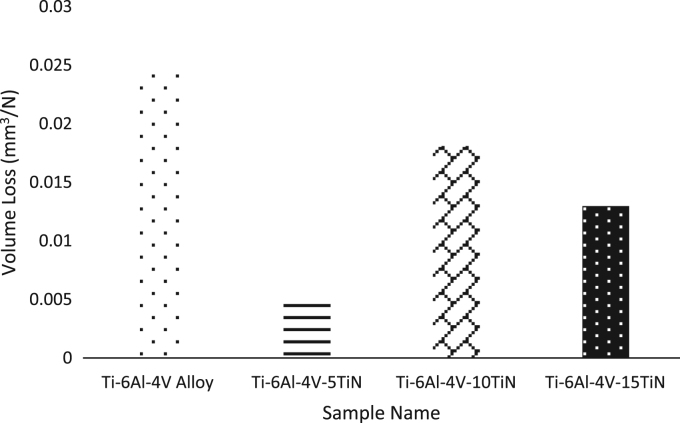
Comparative chart of volume loss of Ti–6Al–4V and Ti–6Al–4V–TiN composites.

Fig. 8 shows the volume loss of the samples after sliding distance of 4 m at normal load of 10 N.

**Fig. 9:**
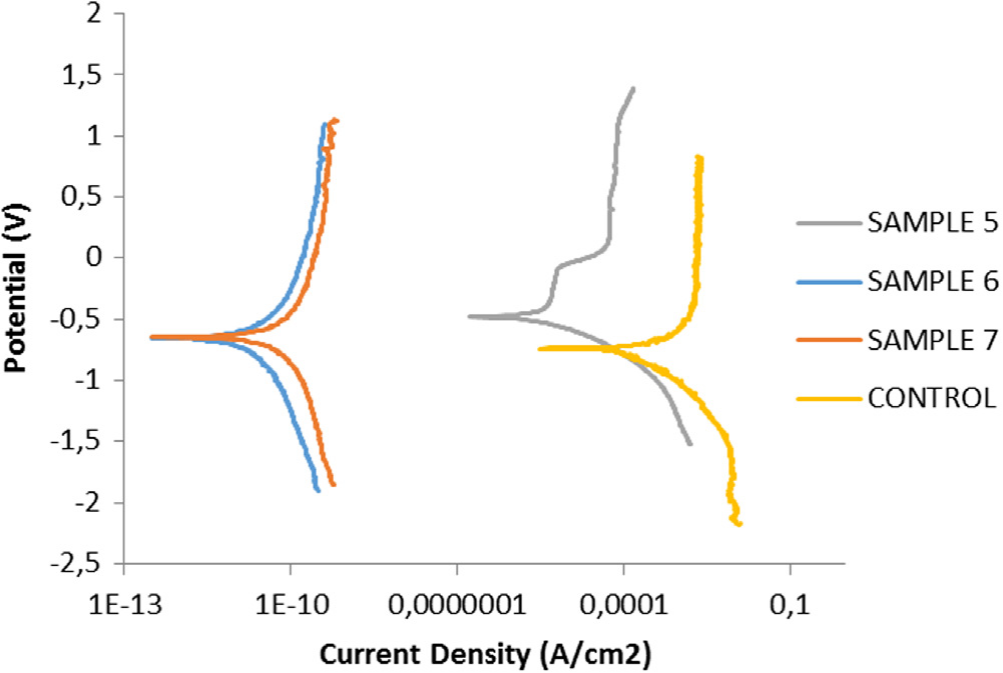
Potentiodynamic polarization curves for Ti–6Al–4V (Control), Ti–6Al–4V–5TiN (sample 5), Ti–6Al–4V–10TiN (Sample 6) and Ti–6Al–4V–15TiN (Sample 7).

Corrosion properties of spark plasma sintered (SPS) Ti–6Al–4V–TiN were explored in 3.65NaCl containing 0.1 M HCl media with the help of potentiodynamic polarization technique [5]. The polarization resistance of the developed compacts is shown in Fig. 9 and Table 3.

**Table 3:** Linear polarization tafel data.

**Sample**	**Ecorr (V)**	**jcorr (A/cm^2^)**	**Corrosion rate (mm/year)**	**Polarization resistance (Ω)**
Ti–6Al–4V Alloy	−0.9463	3.17E−07	0.986625	989
Ti–6Al–4V–5TiN	−0.59306	2.2373E−05	0.14313	6210
Ti–6Al–4V–10TiN	−0.66391	9.98E−03	0.243512	5340
Ti–6Al–4V–15TiN	−0.69734	4.55E−03	0.275244	4768
